# In vitro screening of understudied PFAS with a focus on lipid metabolism disruption

**DOI:** 10.1007/s00204-024-03814-2

**Published:** 2024-07-02

**Authors:** Lackson Kashobwe, Faezeh Sadrabadi, Albert Braeuning, Pim E. G. Leonards, Thorsten Buhrke, Timo Hamers

**Affiliations:** 1 Vrije Universiteit Amsterdam, Amsterdam Institute for Life and Environment (A-LIFE), De Boelelaan 1085, 1081 HV Amsterdam, The Netherlands; 2 Department of Food Safety, German Federal Institute for Risk Assessment (BfR), Max-Dohrn-Str. 8-10, 10589 Berlin, Germany

**Keywords:** PFAS toxicity, Lipid metabolism, Lipid transporters, Bile acid synthesis, HEK293T, HepaRG

## Abstract

Per- and polyfluoroalkyl substances (PFAS) are man-made chemicals used in many industrial applications. Exposure to PFAS is associated with several health risks, including a decrease in infant birth weight, hepatoxicity, disruption of lipid metabolism, and decreased immune response. We used the in vitro cell models to screen six less studied PFAS [perfluorooctane sulfonamide (PFOSA), perfluoropentanoic acid (PFPeA), perfluoropropionic acid (PFPrA), 6:2 fluorotelomer alcohol (6:2 FTOH), 6:2 fluorotelomer sulfonic acid (6:2 FTSA), and 8:2 fluorotelomer sulfonic acid (8:2 FTSA)] for their capacity to activate nuclear receptors and to cause differential expression of genes involved in lipid metabolism. Cytotoxicity assays were run in parallel to exclude that observed differential gene expression was due to cytotoxicity. Based on the cytotoxicity assays and gene expression studies, PFOSA was shown to be more potent than other tested PFAS. PFOSA decreased the gene expression of crucial genes involved in bile acid synthesis and detoxification, cholesterol synthesis, bile acid and cholesterol transport, and lipid metabolism regulation. Except for 6:2 FTOH and 8:2 FTSA, all tested PFAS downregulated *PPARA* gene expression. The reporter gene assay also showed that 8:2 FTSA transactivated the farnesoid X receptor (FXR). Based on this study, PFOSA, 6:2 FTSA, and 8:2 FTSA were prioritized for further studies to confirm and understand their possible effects on hepatic lipid metabolism.

**Supplementary Information:**

The online version contains supplementary material available at 10.1007/s00204-024-03814-2.

## Introduction

Per- and polyfluoroalkyl substances (PFAS) are chemical compounds with advantageous physical and chemical characteristics, making them useful in products that resist water, stains, heat, and biodegradation. They also act as surfactants commonly found in electronics, firefighting foam, food packing, textiles, and cosmetics (Herzke et al. 2012; Kotthoff et al. 2015; Ye et al. 2015). In spite of their potential benefit, in vitro studies reveal that PFAS can bind to lipids, accumulate within the cells (Sanchez Garcia et al. 2018), and disrupt lipid metabolism (Behr et al. 2020b; Louisse et al. 2020). In rodents, several PFAS display strong hepatotoxic effects, which include hypertrophy, vacuolization, necrosis, and liver tumors, which are attributed to PFAS-mediated peroxisome proliferator-activated receptor alpha (PPARα) activation at the molecular level (ATSDR 2021; Dewitt et al. 2012; Dong et al. 2009; Lau et al. 2006). Moreover, in humans, several epidemiological studies consistently revealed that PFOS, PFOA, PFNA, and PFHxS are associated with increased cholesterol and alanine aminotransferase (ALT) levels in serum and decreased immune response (EFSA CONTAM Panel et al. 2020; Frisbee et al. 2010; Grandjean 2018; Steenland et al. 2009; Zhang et al. 2023a, b, c).

PFAS are distinguished by their hydrophobic perfluorinated tail and specific functional groups, and they are commonly categorized according to the type of functional group they possess (Almeida et al. 2021; Buck et al. 2011). For example, perfluoroalkyl carboxyl acids (PFCA) include PFPeA, PFPrA, and PFOA. Perfluoroalkyl sulfonic acids (PFSA) include PFOS. Perfluoroalkyl sulfonamides include PFOSA; fluorotelomer sulfonic acids include 6:2 FTSA and 8:2 FTSA; and fluorotelomer alcohols include 6:2 FTOH (Buck et al. 2011; Scheringer et al. 2014). The type of PFAS' functional group and chain length plays a pivotal role in their hepatotoxicity, with potency variations (Almeida et al. 2021; Rosenmai et al. 2018; Zhang et al. 2023a, b, c; Zhang et al. 2014).

PFAS engage in essential interactions with proteins, including transporters and nuclear receptors (NR), through hydrophobic interactions (Bjork and Wallace 2009). These interactions are crucial for PFAS to form complexes with biological molecules like fatty acids, albumin, or nuclear receptors (Ng and Hungerbuehler 2015; Salvalaglio et al. 2010). For instance, fatty acid activates the PPARα nuclear receptor, forming a complex with the retinoid X receptor (RXR). This PPARα–RXR complex binds to peroxisome proliferator hormone response elements (PPREs), regulating genes related to fatty acid beta-oxidation (Hong et al., 2019; Todisco et al. 2022). Notably, PFAS, like PFOA and PFOS, have exhibited an affinity for the PPARα nuclear receptor activation (Behr et al. 2020a; Buhrke et al. 2015).

PFAS pose a substantial threat to human health due to their bioaccumulating potential and slow renal excretion (DeWitt 2015), with exposure primarily occurring via ingestion and inhalation routes. As indicated in the EFSA Scientific Opinion of 2020, the established tolerable weekly intake for the cumulative sum of PFOA, PFOS, PFHxS, and PFNA was set at PFAS 4.4 ng/kg body weight/week based on the endpoint of immune suppression (EFSA CONTAM panel 2020). Also highlighted in EFSA Opinion 2020 is the association of legacy PFAS to elevated serum cholesterol levels in epidemiological studies. The EFSA Opinion indicates the need for further comprehensive investigation into the molecular mechanism of toxicity of legacy PFAS and alternatives.

**Fig. 1:**
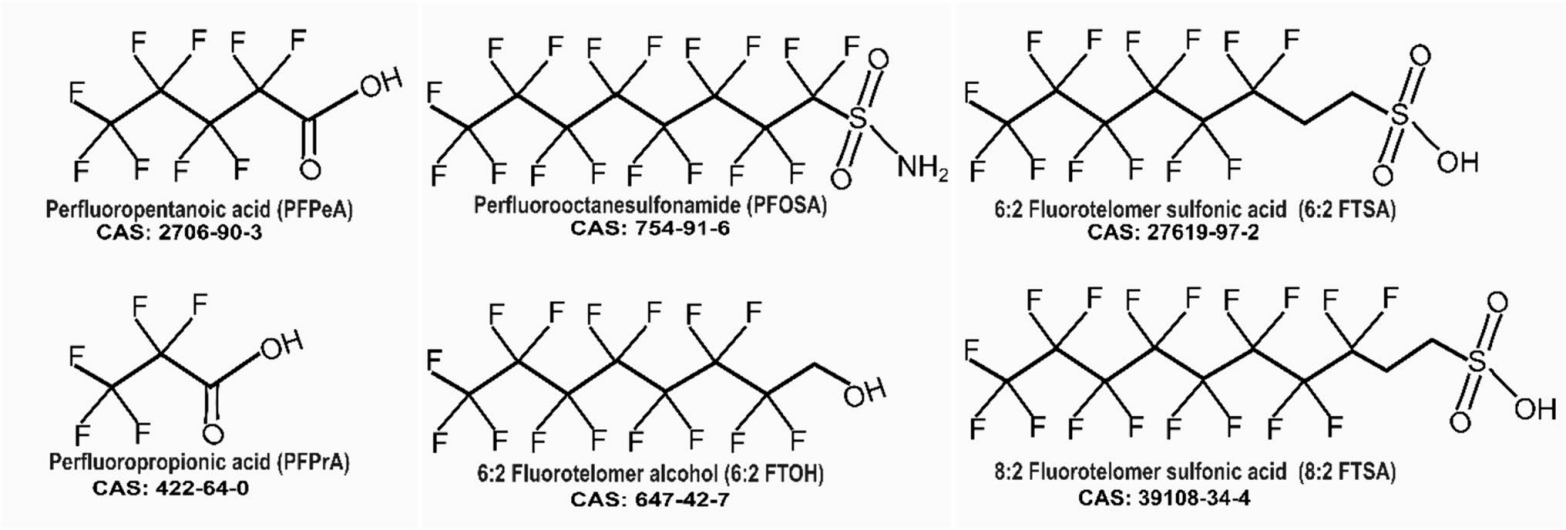
Structure of the studied PFAS. PFPrA and PFPeA belong to perfluorinated carboxylates (PFCA), PFOSA to the perfluorinated sulfonamides, 6:2 FTOH to the fluorotelomer alcohols, 6:2 FTSA, and 8:2 FTSA are classified as fluorotelomers sulfonic acids

The PFAS family comprises nearly 10,000 substances today (Schiavone &and Portesi 2023), and little is known about their toxicity profile. In this study, the focus is on PFPrA and PFPeA short carbon chain (C3, C5) PFAS, and 6:2 FTOH, 6:2 FTSA, 8:2 FTSA, and PFOSA long carbon chain (C8, C10) PFAS (Fig. 1); which their toxicity data is very limited. Most PFAS toxicity data is available for legacy PFAS (PFOA, PFOS, PFNA, and PFHxS), as highlighted in EFSA 2020 opinion. The selection of these PFAS (PFOSA, PFPeA, PFPrA, 6:2 FTOH, 6:2 FTSA, and 8:2 FTSA) for this study was based on their presence in the environment (ATSDR 2021; Washington and Jenkins 2015). These compounds have been found in various sources: For example, PFPeA, PFPrA, and 6.2/8.2 FTSA have been found in drinking water (Chow et al. 2021; Sun et al. 2016; Zheng et al. 2023; Boiteux et al. 2016, 2017), 6.2 FTOH in clothing (van der Veen et al. 2020) and the blood of ski wax technician (Nilsson et al. 2013), and PFOSA in mouthwash, sunscreen, and lip balm (Thépaut et al. 2021). PFPeA has also been found in human samples with increasing concentration overtime (Chang et al. 2021). The toxicity profile of these PFAS are concerning: PFPrA has been shown to cause liver damage and decreased total cholesterol in rats (Lambert et al. 2023), and the potential of 6.2 FTOH to cause hepatocellular necrosis in rats (Rice et al. 2020; Serex et al. 2014), and PFOSA to causes hepatoxicity and disrupt lipid transport in zebrafish (Dasgupta et al. 2020; Xuan et al. 2024). In addition, 6.2 FTSA has been found to cause liver weight increase, necrosis, and inflammation in mice (Sheng et al. 2017). Reardon et al. (2021), using the hepatocyte three-dimensional (3D) cell models, have investigated the toxicity potential of PFPeA, PFOSA, 6.2 FTOH, 6.2 FTSA, and 8.2 FTSA. This study found that PFOSA, 6.2 FTOH, and 8.2 FTSA caused differential expression of hepatocyte genes, with PFOSA being more potent than PFOS.

Based on these findings, our study aimed to screen these six understudied PFAS (Fig. 1) for their capacity to activate nuclear receptors and to cause differential expression of genes involved in hepatic lipid metabolism. The focus on hepatic lipid metabolism was based on previous studies by Behr et al. (2020a; b), who demonstrated that PFOS and PFOA disturb lipid metabolism and activate nuclear receptors PPARα and PPARγ. In our study, the results from reporter gene assays (indicative of nuclear receptor activation) and qPCR measurements (indicative of differential gene expression) are used to prioritize the six PFAS for further studies and to build hypotheses on possibly disrupted pathways in lipid metabolism that might be of interest in such future studies.

## Materials and methods

### Chemicals

PFAS chemicals (Fig. 1) were purchased from Sigma Aldrich, i.e., perfluoro propanoic acid (PFPrA) (> 90%, CAS 422-64-0), perfluoropentanoic acid (PFPeA) (> 90%, 2706-90-3), perfluoro octane sulfonamide (PFOSA) (> 90%, 754-91-6), fluorotelomer sulfonic acids 8:2 FTSA (> 90%, 39108-34-4) and 6:2 FTSA (> 90%, 27619-97-2), and 6:2 fluorotelomer alcohol (6:2 FTOH) (> 90%, 647-42-7). SR 12813 (purity ≥ 98%), troglitazone (purity ≥ 98%), and GW7647 (purity ≥ 98%) were purchased from Cayman (Ann Arbor, USA). CITCO (purity > 99%), GW 4064 (purity ≥ 97%), and GW 501516 (purity ≥ 98%) were purchased from Tocris Bioscience (Bristol, UK). Cyclosporine A (CSA) (purity 99%) and a mixture of palmitic acid and oleic acid, including rosiglitazone (purity > 98%) and dimethyl sulfoxide (DMSO) were obtained from Sigma Aldrich (Taufkirchen or Darmstadt, Germany). Plasmids pGAL4/DBD–CAR/LBD(+ 3aa) (Kanno and Inouye, 2010), pGAL4–hPPARα–LBD, pGAL4–hPPARγ–LBD and pGAL4–hPPARδ–LBD (Kliewer et al., 1997) were kindly provided by Dr. Yuichiro Kanno (Toho University, Funabashi, Japan) and Dr. S. Kliewer (the University of Texas Southwestern Medical Center, Dallas, USA). Plasmids pGAL4–FXR–LBD, pGAL4–LXRα–LBD (Luckert et al. 2018), pGAL4–(UAS) 5-TKLuc, pGAL4–PXR–LBD, pcDNA3–Rluc (Luckert et al. 2013) and pCMXGAL4–hRARα and pCMX–GAL4–hRXRα (Forman et al. 1995) were described previously by Behr et al. (2020a).

### Cell culture

The human hepatic cell line (HepaRG, HPR101) was obtained from Biopredic International (Saint Grégoire, France). The human kidney cell line (HEK293T) was purchased from the European Collection of Authenticated Cell Cultures (Porton Down, UK).

HepaRG cells were seeded and cultured according to Biopredic's cell culture protocol. Briefly, HepaRG cells were cultured in a proliferation medium consisting of William's E medium with 2 mM glutamine (PAN-Biotech, Aidenbach, Germany) supplemented with insulin (5 μg/mL; PAN-Biotech, Aidenbach, Germany), fetal bovine serum (10% v/v; FBS, PAN-Biotech, Aidenbach, Germany), penicillin (100 U/mL) and streptomycin (100 μg/mL) (P/S; Capricorn Scientific, Ebsdorfergrund, Germany), and hydrocortisone hemisuccinate (50 μmol/L HHS; Sigma-Aldrich, St. Louis, USA) for 14 days to proliferate. After 14 days, the HepaRG cells were cultured in a proliferation medium containing 1% DMSO (> 99% purity, Sigma Aldrich) (v/v) for 2 days, followed by a proliferation medium supplemented with 1.7% DMSO (v/v) as a differentiation medium for up to day 28 to generate fully differentiated hepatocytes. After 28 days, the HepaRG cell medium was changed to a treatment medium consisting of William's E medium as a basal medium supplemented with 2% FBS, 100 U/mL penicillin, 100 μg/mL streptomycin (P/S), hydrocortisone hemisuccinate (50 μmol/L), and 0.5% DMSO for 2 days. The cells were incubated at 37 °C in a humidified atmosphere with 5% CO_2_. The medium was changed every 2–3 days. The fully differentiated HepaRG cells were exposed to various PFAS concentrations prepared in a treatment medium for 24 h, with a final 0.5% DMSO concentration.

HEK293T cells were seeded at 2 × 10^4^ cells/well in 96 well plates and cultured using the manufacturer's protocol. Briefly, HEK293T cells were grown in Dulbecco's modified Eagle's medium (DMEM, PAN-Biotech, Aidenbach, Germany) supplemented with 10% (v/v) fetal bovine serum (FBS, PAN-Biotech, Aidenbach, Germany), and 100 U/mL penicillin and 100 μg/mL streptomycin (P/S, Capricon Scientific, Ebsdorfergrund, German). The cells were kept at 37 °C in a humidified atmosphere with 5% CO_2_ and were exposed to different PFAS concentrations diluted in DMEM with 0.5% DMSO concentrations for 24 h.

### Cytotoxicity assay

The cytotoxicity of the tested compounds was determined by 3-(4,5-dimethyl-thiazol-2-yl)-2,5-diphenyltetrazolium bromide (MTT) assay as described by Scharmach et al. (2012). Briefly, using a 96 well-plate, HEK293T and differentiated HepaRG cells were exposed to PFOSA, PFPrA, PFPeA, 6:2 FTOH, 6:2 FTSA, and 8:2 FTSA in concentration series (1, 10, 25, 50, 100, 250, 500, and 1000 µM) for 24 h. One hour before the end of the exposure period, 10 µL MTT reagent undiluted was added to the PFAS incubated wells. After exposure, plates were centrifuged, and the medium was aspirated. Afterward, 130 µL MTT desorption solution was preheated at 37 °C and added to the wells. The plate was placed on a shaker (600 rpm) at room temperature for 30 min, and after that, the absorption was measured at 570 nm. The assay was performed in quadruplicate with three biological replicates. Untreated cells served as a negative control, and 0.01% Triton X-100 (Sigma Aldrich) was used as a positive control.

### Nuclear receptor transactivation assay

The impact of PFOSA, PFPrA, PFPeA, 6:2 FTOH, 6:2 FTSA, and 8:2 FTSA on the promoter activity of different nuclear receptors was determined by the luciferase reporter gene assay as described by Hampf and Gossen (2006). Briefly, HEK293T cells were transiently transfected with the plasmid for the expression of the GAL4–LBD construct of the respective human nuclear receptor (hFXR, hPXR, hLXRα, hRARα, hPPARα, hPPARγ, hPPARẟ, and hCAR), and co-transfected with the GAL4-dependent luciferase reporter plasmid pGAL-(UAS)_5_-TK-luc and the *Renilla* luciferase construct pcDNA3–Rluc for normalization using the TransIT–LT1 transfection reagent (Mirus Bio, Madison USA) according to the manufacturer's protocol. After transfection, the cells were exposed for 24 h to 1, 10, 25, 50, 100, 250, and 500 µM concentrations of PFPeA, PFPrA, 6:2 FTOH, and 8:2 FTSA; 1, 2.5, 5, 10, 25, 50, and 100 µM concentrations of PFOSA, and 1, 5, 10, 25, 50, 100, and 250 µM concentrations of 6:2 FTSA. An agonist for the respective nuclear receptor was used as a positive control in each reporter gene assay, i.e., GW4064 for hFXR, SR12813 for hPXR, GW3965 for hLXRα, AM580 for hRARα, GW7647 for hPPARα, troglitazone for hPPARγ, GW501516 for hPPARẟ, and CITCO for hCAR. Experiments were done in three to six technical replicate wells per condition with three biological replicates for the PFAS, which showed nuclear receptor activity.

### Gene expression analysis

HepaRG cells were exposed to different test substances (PFAS) for 24 h. The selected PFAS concentration included 10, 100, and 500 µM for PFPrA, PFPeA, 6:2 FTOH, 6:2 FTSA, and 8:2 FTSA. For PFOSA, 10 and 100 µM concentrations were studied. As positive controls, 20 µM cyclosporin A (CSA), a compound known to induce cholestasis, 200 µM cyproconazole (CYPRO), a fungicide known to cause steatosis in rodents, and 500 µM of a mixture of oleic acid and palmitic acid, inducers of endogenous de novo lipogenesis, were used.

After exposure, cells were washed with ice-cold PBS, and RNA was extracted using the RNeasy mini kit (Qiagen, Hilden, Germany) following the manufacturer's protocol. Total RNA was quantified with a spectrophotometer (Nanodrop 1000, Nanodrop Technologies, Wilmington, USA). The high-capacity cDNA reverse transcriptase kit (Applied Biosystems, Foster City, USA) was used to produce cDNA. Real-time quantitative PCR (qPCR) was performed on a CFX (Bio-Rad, life research sciences) using SYBR Green qPCR Master Mix (Bio-Rad, life research sciences). The thermal conditions were set at 95 °C for 5 min to warm up, followed by 40 cycles of 15 s at 95 °C and 45 s at 60 °C. Relative changes in mRNA transcription levels were quantified using the 2^−ΔΔ*ct*^ method (Pfaffl 2001) normalized to the housekeeping genes GAPDH and GUSB. Three independent biological experiments were performed. Table S1 (supplementary information) presents the list of primers used. The primer selection was based on Behr et al. (2020b) study, in which PFOS and PFOA impacted genes involved in bile acid synthesis, transport and detoxification, cholesterol synthesis and transport, and nuclear receptors regulating lipid metabolism. In addition, primer selection considered steatosis marker genes, based on the study of Lichtenstein et al., (2020).

### Statistics

Results from the reporter gene bioassay and the qPCR were evaluated using one-way analysis of variance (ANOVA) using Dunnett's post-hoc test (*p* < 0.05) to determine differences between the solvent control and treated groups. The statistical analysis was performed on three biological replicates using GraphPad Prism 9.

## Results

### Cytotoxicity

**Fig. 2:**
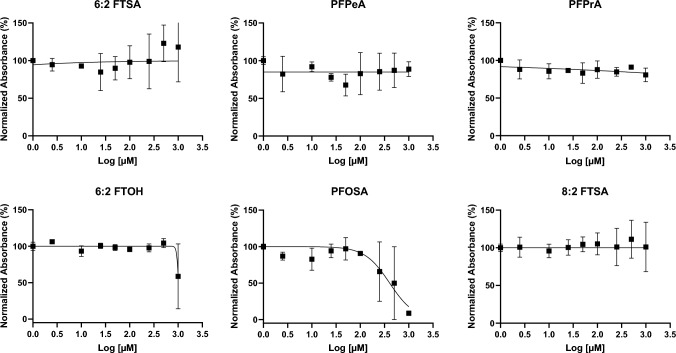
Cytotoxicity profile of the six PFAS based on HepaRG cell viability. The cells were exposed to different concentrations of 6:2 FTSA, PFPeA, PFPrA, 6:2 FTOH, PFOSA, and 8:2 FTSA for 24 h. The cellular viability was measured using the MTT assay. Viability is shown as a percentage (%) relative to solvent control (SC) set at 100%. Experimental data are the mean of the three biological replicates, and error bars show the standard deviation (SD) of biological replicates

Following a 24 h exposure to 6:2 FTSA, PFPeA, PFPrA, and 8:2 FTSA, the HepaRG cells showed no decrease in cell viability in the MTT cytotoxicity assay. On the other hand, 6:2 FTOH reduced cell viability to < 40% at 1000 µM. At 100 µM PFOSA exposure, HepaRG cells showed a 9.52% decrease in cell viability after 24 h, compared to the solvent control (SC). Interestingly, this cell viability was decreased by < 20% at lower concentrations (1 and 10 µM). HepaRG cell viability decreased by > 30% when exposed to > 250 µM PFOSA for 24 h (Fig. 2). In HepaRG cells, the estimated IC50 was 1016 µM for 6:2 FTOH and 402 µM for PFOSA. The observed variation in HepaRG cell viability can be attributed to biological replicates rather than technical replicates. Meanwhile, in HEK293T cells (as shown in Fig. S1), exposure to 500 µM 6:2 FTSA and 250 µM PFOSA led to a decrease in cell viability to < 50%, but 6:2 FTOH only at 1000 µM. The cytotoxicity results for HepaRG and HEK293T aligned closely, except for 6:2 FTSA.

We selected the nontoxic concentrations for the nuclear receptor activation assay and gene expression study based on the cytotoxicity results. In the nuclear receptor activation assays, PFPeA, PFPrA, 6:2 FTOH, and 8:2 FTSA were tested up to 500 μM, 6:2 FTSA up to 250 µM, and PFOSA up to 100 µM. For the gene expression studies, 10, 100, and 500 μM concentrations were used for all PFAS, except for PFOSA, for which the highest test concentration of 500 µM was excluded.

### Nuclear receptor activation

**Fig. 3:**
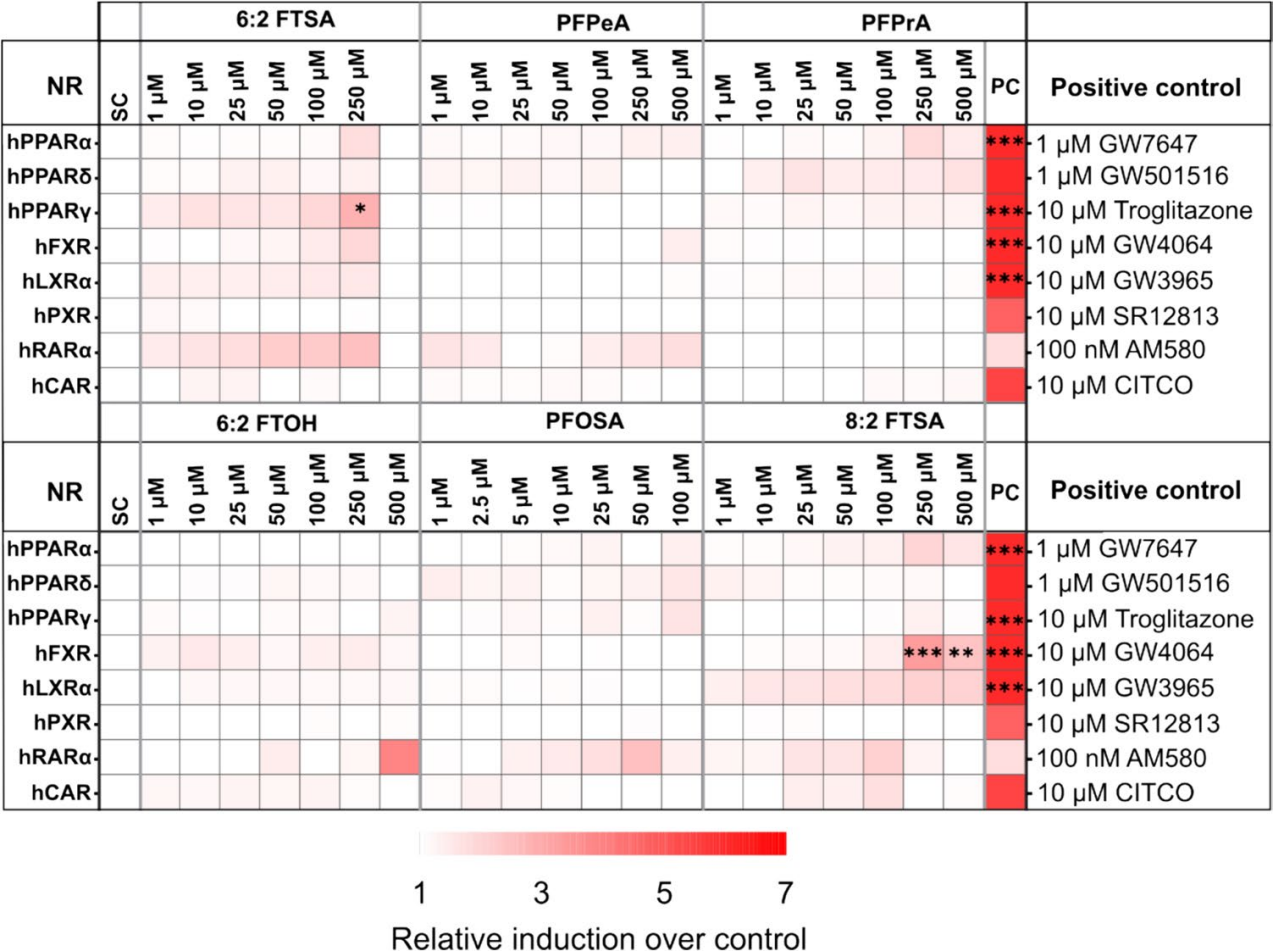
Activation of nuclear receptors by PFAS. Summary of the results of the transactivation assays as a heatmap. The heatmap is based on average biological replicates of experimental data (mean + SD). Statistical analysis was performed on three biological replicates, as indicated by asterisks, ***p < 0.001, **p < 0.01, *p < 0.05; one-way ANOVA with Dunnett's post-hoc test

Luciferase-based reporter gene assays were used to determine PFOSA-, PFPeA-, PFPrA-, 6:2 FTOH-, 6:2 FTSA-, and 8:2 FTSA-mediated activation of the nuclear receptors (PPARα, PPARẟ, PPARγ, FXR, LXRα, PXR, RARα, and CAR), which are involved in the regulation of lipid or xenobiotic metabolism. The results of the transactivation assays are summarized as a heatmap (Fig. 3). The respective positive control strongly activated the different nuclear receptors. In contrast, only slight activation, if at all, was observed for the tested PFAS. None of the tested PFAS activated PPARẟ, PXR, RARα, and CAR. Out of the eight nuclear receptors tested, the reporter gene assays indicated that 8:2 FTSA at 250 µM and 500 µM concentrations had an agonistic effect on FXR. LXRα was slightly activated, although not significantly. 6:2 FTSA showed some slight activation on PPARγ at 250 µM concentration. The statistical analysis was done on three biological replicates, and only two biological replicates were performed for PFAS that did not show any nuclear receptor activation.

### Gene expression

**Fig. 4:**
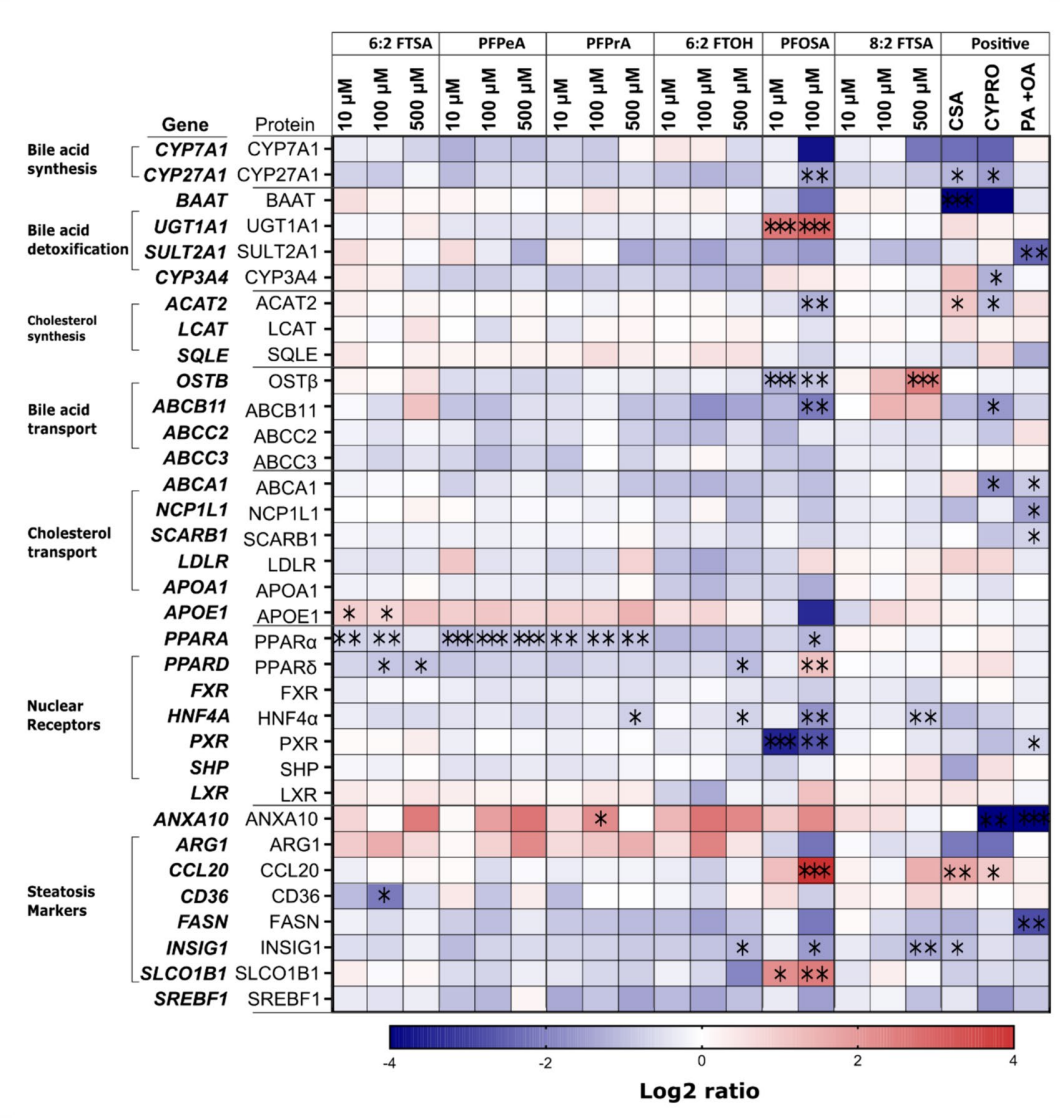
Gene expression study of genes involved in lipid metabolism. HepaRG cells were exposed to varying concentrations of 6:2 FTSA, PFPeA, PFPrA, 6:2 FTOH, PFOSA, 8:2 FTSA, CSA, CYPRO, or palmitic acid (PA)/oleic acid (OA) for 24 h. The mRNA levels were normalized to GADPH and GUSB mRNA expression. The log2 ratio shows the relative gene expression compared to solvent control. Gene expression was measured in three biological replicates and analyzed using one-way ANOVA with Dunnett's post-hoc test. Significance based on *p* value is indicated as asterisks (***p < 0.001, **p < 0.01, *p < 0.05)

In the gene expression study with differentiated HepaRG cells, the positive controls cyclosporin A (CSA) and cyproconazole (CYPRO) consistently revealed a notable decrease in the activity of the majority of the tested genes (Fig. 4). Both CSA and CYPRO displayed a similar gene expression pattern. CSA, a well-known inducer of cholestasis (Hulzebos et al. 2004; Sharanek et al. 2015), downregulated gene expression of *CYP7A1, CYP27A1, BAAT, HNF4A,* and *INSIG1,* while it upregulated *ACAT2* and *CCL20* gene expression*.* CYPRO, a fungicide shown to induce steatosis in rodents (al-Eryani et al. 2015; Marx-Stoelting et al. 2017), decreased gene expression of *CYP7A1, CYP27A1, BAAT, CYP3A4, ACAT2, ABCB11, ABCA1, SCARB1*, *HNF4A*, *PXR*, *ANXA10*, while it upregulated *CCL20* gene expression. Palmitic acid and oleic acid (PA + OA) are known fatty acids that promote endogenous de-novo lipogenesis (Castillo et al. 2023; Murru et al. 2022); they decreased the expression of several genes, including *SULT2A1, ABCA1, NCP1L1, SCARB, PXR, ANXA10,* and* FSN.*

Exposing HepaRG cells to various PFAS led to the downregulation of most genes (Fig. 4; depicted in blue), although not statistically significant in certain instances. PFOSA exhibited distinctive effects on specific genes, notably upregulation of *UGT1A1*, *CCL20*, *PPARD*, *ANXA10*, and *SLCO1B1* gene expression, setting it apart from other tested PFAS and positive controls. Moreover, in PFOSA-exposed HepaRG cells, the most significant gene expression effects were observed at 100 µM concentration. However, at 10 µM concentration, PFOSA upregulated *UGT1A1* and *SLCO1B1* gene expression and downregulated *OSTB* and *PXR* gene expression*.*

6:2 FTSA exhibited varying effects at different concentrations. At 10 and 100 µM, it induced an increase in *APOE* gene expression while causing a decrease in *PPARA* gene expression*.* At 100 and 500 µM concentrations, *PPARD* gene expression was downregulated, with a notable decrease in *CD36* gene expression observed at 100 µM. PFPeA and PFPrA consistently downregulated *PPARA* gene expression across 10, 100, and 500 µM concentrations. In addition, at 500 µM concentration, PFPrA suppressed the expression of the *HNF4A* gene*,* while at 100, it triggered an increase in *ANXA10* gene expression. A 500 µM exposure of HepaRG cells to 6:2 FTOH reduced the gene expression of *PPARD*, *HNF4A*, and *INSIG1*.

## Discussion

Multiple studies, as documented by ATSDR (2021), EFSA (2018), and EFSA CONTAM Panel et al. (2020), have examined the adverse effects of PFOA, PFOS, PFHxS, and PFNA. Collectively, these investigations show the liver as a primary target organ for PFAS-induced toxicity. In rodent liver, PFAS observed effects include weight increase, hyperplasia, hepatocellular hypertrophy, and necrosis (Dewitt et al. 2012; Dong et al. 2009; Zeng et al. 2019). While many of these PFAS-induced effects are posited to be PPARα-dependent at a molecular level, alternative mechanisms contributing to hepatoxicity have been documented and exemplified in PPARα-null mice (ATSDR 2021; Bjork et al. 2011; Rosen et al. 2008).

The central aim of this study was to screen six understudied PFAS (PFOSA, PFPeA, PFPrA, 6:2 FTOH, 6:2 FTSA, and 8:2 FTSA) for their capacity to activate nuclear receptors and their impact on gene expression related to lipid metabolism. The outcome of this study will be used to prioritize PFAS for further studies and build hypotheses on possibly disrupted pathways in lipid metabolism to be addressed in such studies.

Results from the reporter gene assays indicated activation of nuclear receptors FXR by 8:2 FTSA and PPARγ by 6:2 FTSA. None of the tested PFAS exhibited significant activation of PPARα, which is noteworthy because most PFAS previously investigated have shown PPARα activation (Behr et al. 2020a). Slight PPARα activation was observed for PFOSA at 100 µM and all other selected PFAS at concentrations ≥ 250 µM except for 6:2 FTOH; however, these activations were not statistically significant (Fig. 3). Moreover, no activation was observed for PPARδ, PXR, RARα, and CAR, aligning with prior studies on nuclear receptor activation by PFCAs and PFSAs (Abe et al. 2017; Behr et al. 2020a). PPARα and PPARγ serve as lipid metabolism regulators by promoting fatty acid beta-oxidation and fatty acid storage, respectively (Glatz & Luiken 2018; Schwenk et al. 2010; Hong et al. 2019; Tyagi et al. 2011). FXR serves as a regulator of bile acid synthesis and cholesterol homeostasis by suppressing the transcriptional activities of LXR and HNF4α (Alaynick 2008; Becnel et al. 2015; McKenna et al. 2014; Shin & Wang 2019; Tata 2002).

Studies by Almeida et al. (2021) and Zhang et al. (2014) revealed that nuclear receptor activation by PFAS is chain-length dependent. Also, a molecular docking and modeling study on PFAS-PPARγ receptor interaction showed that tyrosine (Tyr) 473 amino acid is crucial in activating PPARγ (Almeida et al. 2021). In the same study, 6:2 FTOH, 6:2 FTSA, and PFOSA did not interact with Tyr 473, resulting in a non-activation of PPARγ. This study corroborates our findings that 6:2 FTOH and PFOSA do not activate PPARγ. However, 6:2 FTSA at 250 µM showed a twofold increase in PPARγ activity compared to control. In this study, our reporter gene assay results indicate that 6:2 FTSA and 8:2 FTSA may affect the metabolism of fatty acid, cholesterol, and bile acid.

Among the six tested PFAS, PFOSA, a precursor of PFOS (Chen et al. 2022, 2015; Wang et al. 2013, 2017), showed the highest cytotoxicity towards HepaRG cells. This result aligns with previous findings on cytotoxicity of the same PFAS, excluding PFPrA (Reardon et al. 2021). PFOSA also had more effects on transcriptional activity on tested gene expression involved in lipid metabolism than all six tested PFAS. The impact of PFOSA on genes associated with lipid metabolism is comparable to that of PFOS (Behr et al. 2020b; Louisse et al. 2020; Reardon et al. 2021). This finding suggests that the HepaRG cells, a hepatic cell line with high metabolizing capacity, may convert PFOSA to PFOS. Thus, the observed effects of PFOSA might be due to PFOS-mediated impacts. On the other hand, the sulfonamide functional group of PFOSA itself might also contribute to the observed effects, as shown in a study by Rericha et al. (2022) in zebrafish embryos.

PFOSA decreased the gene expression of *PPARA* while it upregulated the expression of the *PPARD* gene. *PPARA* and *PPARD* genes encode for PPARα and PPARδ receptor proteins, respectively, which promote the oxidation of fatty acid, glucose, and amino acid metabolism (Liu et al. 2018; Rakhshandehroo et al. 2007; Strosznajder et al. 2021; Berthier et al. 2021). Repressing the expression of the *PPARA* gene can cause less PPARα, reducing FA beta-oxidation and ketogenesis, which may increase fatty acid storage as triglyceride. In the same HepaRG cells exposed to PFOSA, PPARα-dependent genes such as *CYP7A1, CYP27A1*, and *ACAT2* (Kersten et al. 2010; Rakhshandehroo et al. 2007; Todisco et al. 2022) were downregulated. PPARα is crucial in preventing the likelihood of developing metabolic-related diseases by decreasing triglyceride levels (fat storage) (Glatz and Luiken 2014, 2018; Hernandez-Quiles et al. 2021). PPARδ is essential in modulating cellular energy consumption, sensitivity, and insulin secretion (Liu et al. 2018; Li and Glass 2004). Overexpression of the *PPARD* gene could, similarly to the downregulation of the *PPARA* gene, significantly disrupt lipid metabolism by impairing fatty acid beta-oxidation and synthesis (Higgins et al. 2012). PPARδ overexpression also promotes inflammation and tumorigenesis (Bougarne et al. 2018; Liu et al. 2018; Pawlak et al. 2015; Tyagi et al. 2011). Therefore, the upregulation of *PPARD* gene expression could also indicate a pro-inflammatory response due to PFOSA effects. Moreover, PFOSA-treated cells overexpressed the *CCL20* gene, which encodes for the chemokine CCL20 that recruits immune cells to the liver and plays a crucial role in immunomodulatory and inflammatory responses (Kwantwi et al. 2021). The *CCL20* gene is also implicated in steatosis adverse outcome pathways (AOP) (Lichtenstein et al. 2020).

PFOSA suppressed the gene expression of *HNF4A*, which encodes for the HNF4α protein essential in modulating bile acid metabolism through the activation of *CYP7A1/27A1* gene expression (Shin and Wang 2019; Zhang & Chiang 2001). Also, HNF4α regulates the expression of genes involved in bile acid transport (*SLCO1B1* and *ABCB11*) and detoxification (*BAAT*) (Halilbasic et al. 2013; Yin et al. 2011). The repression of *HNF4A* gene expression in PFOSA-exposed HepaRG cells may have caused the observed decrease in the expression of *ABCB11*, *CYP27A1*, and *CYP7A1* genes*.* Previous studies in HepaRG cells (Behr et al. 2021) and in HepG2 cells (Beggs et al. 2016; Scharmach et al. 2012; Buhrke et al. 2015) revealed that PFOS and PFOA also downregulated *HNF4A* gene expression. A decrease in HNF4α may significantly reduce bile acid and cholesterol synthesis and transportation (Won et al. 2019). Furthermore, expression of the *PXR* gene was downregulated by PFOSA, possibly due to decreased gene expression of *HNF4A* and *PPARA.* The *PXR* gene codes for PXR, a ligand-activated nuclear receptor that modulates the inducible expression of target genes associated with xenobiotic metabolism, e.g., *CYP3A4* and *UGT1A1* gene (Brewer and Chen 2016; Kandel et al. 2016; Pavek 2016). PPARα and HNF4α can heterodimerize with PXR to regulate genes involved in lipid, cholesterol, and bile acid metabolism (Pavek 2016), indicating that changes in the expression of one nuclear receptor can have an impact on the expression and functioning of other nuclear receptors (Higgins et al. 2012; Kasano-Camones et al. 2023)*.*

Bile acid biosynthesis is mainly regulated by CYP7A1, an enzyme in the first rate-limiting step in the classical bile acid (BA) formation pathway. CYP27A1 is also involved in bile acid production. Both enzymes catalyze the hydroxylation of cholesterol to produce intermediate molecules in bile acid formation (Sawada et al. 2001). Gene expression results showed that PFOSA downregulated the expression of *CYP7A1* and *CYP27A1* genes, although not statistically significant for *CYP7A1*. Also, PFOSA upregulated the gene expression of *UGT1A1*, which encodes for a protein (UGT1A1) essential in detoxifying toxic substances and in the glucuronidation of bilirubin, a necessary step in bile acid excretion (Claudel et al. 2011). The effect of PFOSA on the differential gene expression of *CYP7A1*, *CYP27A1*, and *UGT1A1* could ultimately affect bile acid production, conjugation, detoxification, and excretion.

**Fig. 5:**
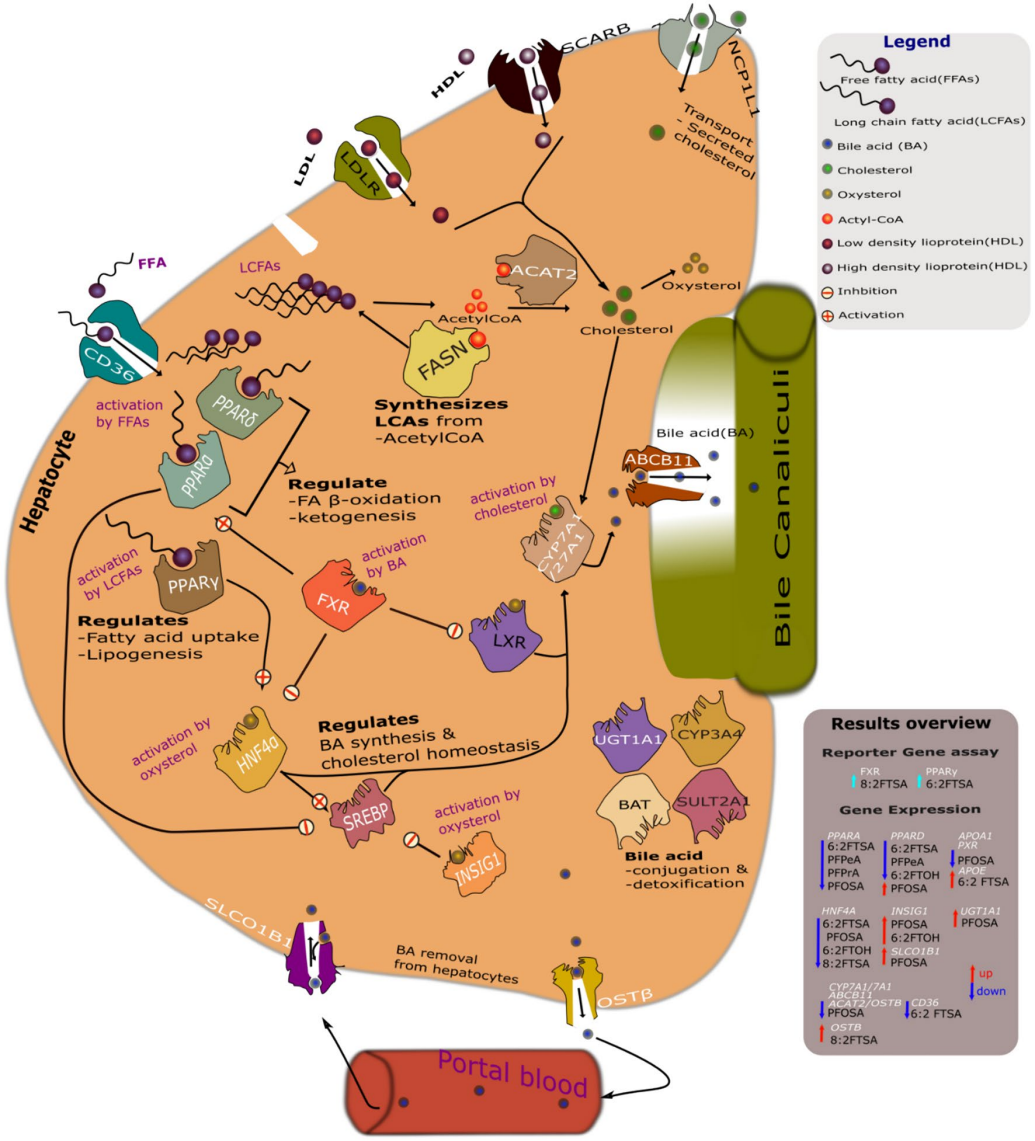
Pathways involved in lipid metabolism according to literature (see text for references). The figure illustrates the role of selected proteins of interest (encoded by their respective genes; see Fig. 4) in lipid uptake and degradation via fatty acid beta-oxidation and de novo lipogenesis. The results overview (to the right) indicates which PFAS causes nuclear receptor activation (cyan arrows), gene downregulation (blue arrows), or upregulation (red arrows). Significantly affected genes are written in white, and PFAS are written in black. Fatty acid (FA) and long carbon fatty acid (LCFAs) are initially transported into hepatocytes via the CD36 receptor. The available FAs are converted to acetyl CoA and esterified by ACAT2 to generate cholesterol. Low-density lipoprotein (LDL) and high-density lipoprotein (HDL) containing cholesterol are carried into hepatocytes via LDL receptor (LDLR) and SCARB1, respectively. FAs and LCFAs activate PPARα and PPARγ, respectively. PPARα regulates FA beta-oxidation and ketogenesis and blocks the activities of SREBP, which regulates bile acid (BA) biosynthesis and cholesterol homeostasis. PPARγ modulates FA uptake and lipid synthesis, and its activation promotes the expression of the HNF4α, which regulates cholesterol metabolism and lipid homeostasis. The HNF4α expression promotes the expression of CYP7A1/27A1, which converts cholesterol into bile acid (BA). The oxysterols, a by-product of cholesterol oxidation, trigger the expression of the INSIG1 and HNF4α. INSIG1 inhibits the transcriptional activities of SREBP in response to high bile acid and cholesterol levels. The activation of the FXR nuclear receptor by BA represses the activation of HNF4α and LXR. SLCO1B1 transports BA to and from portal blood, NCP1L1 transports cholesterol into the Hepatocyte, ABCB11 carries BA into the canaliculus, and OSTβ pumps BA into the circulation (colour figure online)

Bile acid accumulation can activate the canalicular export system, which comprises several genes responsible for bile acid transport across the cell membrane, including *ABCB11*, *SLC51B (OSTB)*, and *SLCO1B1* (Garrison et al. 2020; Halilbasic et al. 2013; Li and Chiang 2013; Rao et al. 2007; St-Pierre et al. 2001). In PFOSA-treated cells, the expression of the *SLCO1B1* gene was upregulated, while the expressions of the *ABCB11* and *OSTB* genes were downregulated. ABCB11 pumps bile acid into the bile canaliculi, OSTβ removes bile acid from hepatocytes into portal blood as an alternative route, and SLCO1B1 (or OATP1B1) transports bile to and from the portal blood (Mahagita et al. 2007; Telbisz and Homolya 2016; Jonker et al. 2009; Shin and Wang 2019) (Fig. 5). SLCO1B1 is identified as a steatosis marker (Lichtenstein et al. 2020). The decreased expression of *OSTB* and *ABCB11* genes may be due to low levels of bile acid formation due to suppression of *HNF4A, CYP7A1,* and *CYP27A1* gene expression. Moreover, inhibiting OSTβ and ABCB11 transporters can disrupt the bile flow out of the hepatocyte and into the canaliculi, leading to bile acid accumulation inside hepatocytes. The alteration in bile flow can cause intrahepatic cholestasis, a liver disease that occurs when bile flow is reduced or blocked (Halilbasic et al. 2013; Jonker et al. 2009). On the other hand, the upregulation of SLCO1B1 expression can compensate for the deficiency in OSTβ and ABCB11 function (Mahagita et al. 2007).

PFOSA also reduced the expression of the *APOA1* and *ACAT2* genes. *APOA1* gene encodes for apolipoprotein A1 (APOA1), a structural protein in lipoproteins that plays a crucial role in reverse cholesterol transport (Blake et al., 2021). APOA1 interacts with different receptors, such as ATP-binding cassette transporter A1 (ABCA1) and Scavenger receptor class B type 1 (SCARB1) (Xu et al. 2022). The *ACAT2* gene produces an enzyme (ACAT2) responsible for synthesizing cholesterol from cholesteryl esters and long-chain fatty acids (Bell et al. 2006; Pramfalk et al. 2008). The decrease in *APOA1* gene expression may be due to inhibited enterohepatic cholesterol transport or low cholesterol levels resulting from decreased *ACAT2* gene expression. Suppressed *ACAT2* gene expression may affect bile acid formation, as evidenced by the downregulation of *CYP7A1/27A1* and *HNF4A* gene expression. Figure 5 shows the roles that the proteins encoded by the genes examined in this study have in different hepatic metabolism processes, according to what has been reported in the literature. Changes in the levels or activities of these proteins may have affected different lipid metabolism processes, as can be hypothesized from Fig. 5.

The gene expression results also revealed that 6:2 FTSA downregulated the expression of *PPARA*, *PPARD,* and *CD36* genes. As discussed for PFOSA before, *PPARA* and *PPARD* genes regulate fatty acid metabolism. *CD36* gene encodes for a transmembrane protein transporting fatty and long-chain fatty acids into cells (Berthier et al. 2021; Maréchal et al. 2018). In the reporter gene assay, 6:2 FTSA shows a slight activation of the PPARy nuclear receptor, which also induces the transcription of the CD36 transporter (Li & Glass 2004). The transactivation of PPARy in the reporter gene assay, in combination with the decreased expression of the *CD36* gene, suggests that 6:2 FTSA may have a PPARy-independent inhibitory role on *CD36* gene expression. Also, 6:2 FTSA increased *APOE* gene expression. APOE is a lipoprotein component that aids in transporting cholesterol via low-density lipoprotein receptors (LDLR) into the cell and modulates lipid and lipoprotein homeostasis (Getz and Reardon 2009; Martins et al. 2006). These findings suggest increased cholesterol transportation into the cell, and APOE overexpression can enhance cellular lipid accumulation (Pawlak et al. 2015).

8:2 FTSA downregulated *HNF4A* and *INSIG1* gene expression, while in the reporter gene assay, it activated FXR, which is crucial in regulating bile acid metabolism and maintaining homeostasis (Calkin and Tontonoz 2012). FXR activation switches off the transcriptional activity of HNF4α and LXR but increases the expression of OSTβ and ABCB11 transporters (Jonker et al. 2009; Zhang and Chiang 2001), as was also observed in the present study, albeit not statistically significant for *ABCB11*. In addition, the suppression of HNF4α can cause a decrease in the expression of the *SREBP* gene, which promotes cholesterol synthesis. Moreover, 8:2 FTSA repressed the *INSIG1* gene, which encodes for a reticulum membrane protein (INSIG1), regulating cholesterol synthesis and lipogenesis by binding to SREBP in a negative feedback mechanism (Azzu et al. 2021; Carobbio et al. 2013; Engelking et al. 2005).Taken together, 8:2 FTSA promotes gene expression of bile acid transporters *OSTB* and *ABCB11* presumably via FXR activation and, in addition, has an inhibitory effect on gene expression of cholesterol regulators *INSIG1* and *HNF4A*.

Other tested PFAS downregulated gene expression of *PPARA* (PFPeA and PFPrA), *HNF4A* (PFPrA and 6:2 FTOH)*,* and *INSIG1* (6:2 FTOH).

Several studies suggest that exposure to toxic chemicals can cause and accelerate steatosis, known as toxicant-associated fatty liver disease (TAFLD) (AbdulHameed et al. 2019; al-Eryani et al. 2015; Kaiser et al. 2012; Negi et al. 2021). Hepatic steatosis is the accumulation of excess fat, such as triglycerides, in hepatocytes and is commonly associated with alcoholic and nonalcoholic fatty liver diseases (Nassire et al. 2015). So far*, ARG1, FASN, INSIG1, SLCO1B1,* and *SBREBF1* genes (Lichtenstein et al. 2020), and *PPAR* genes (AbdulHameed et al. 2019) have been identified as potential contributors to lipid accumulation. According to the proposed AOP for fatty liver-related diseases by Mellor et al. (2016), ten ligand-activated transcription factors, i.e., LXR, AhR, PXR, PPARα, PPARγ, FXR, CAR, PPARẟ, RAR, glucocorticoid receptor (GR), and estrogen receptor (ER) are involved in the fatty liver-related diseases. In our study, most of these genes were repressed by PFAS. For example, PFOSA, 6:2 FTOH, and 8:2 FTSA decreased *INSIG1* gene expression, although *PFOSA* upregulated *SLCO1B1* gene expression. 6:2 FTSA, PFPeA, PFPrA, and PFOSA decreased the gene expression of *PPARA* and *PPARD.* PFOSA upregulated the gene expression of *PXR* whereas, in the reporter gene assay, 6:2 FTSA and 8:2 FTSA increased the transcriptional activities of PPARγ and FXR nuclear receptors, respectively.

PFPrA induced the gene expression of the *ANXA10*; although statistically insignificant, increased ANXA10 gene expression trends were also observed in cells exposed to 6:2 FTSA, PFPeA, 6:2 FTOH, and PFOSA. ANXA10 is crucial in apoptosis, vesicle trafficking, calcium signaling, growth control, and cell division (Moss and Morgan 2004; Zhang et al. 2023a, b, c), and it is a candidate marker for cancer progression, diagnosis, and prediction (Tsai et al. 2015; Zhang et al. 2023a, b, c).

Results from the current screening study can be used to prioritize PFAS for future in-depth studies, but should also be interpreted with care. The observed nuclear receptor activation and differential gene expression can pinpoint the direction of such future studies (Fig. 5), but the hypothesized changes in protein activities and lipid metabolism pathway require further confirmation. Moreover, the concentration range tested in this study (10–100 µM) is three to four orders of magnitude higher than physiologically relevant concentrations, which are in a range of about 10 nM for PFOS and PFOA in serum (D'Eon and Mabury 2011; Roth et al. 2020). Nevertheless, this study provides novel in vitro data on the impact of understudied PFAS on nuclear receptor activity and gene expression related to lipid metabolism in the in vitro HepaRG hepatocyte model, with additional knowledge on their cytotoxicity.

## Conclusion

Our findings highlight PFOSA as the most potent among the six tested PFAS and capable of interfering with pathways essential for lipid synthesis, degradation, storage, and bile acid synthesis and transport similar to PFOS. In addition, 8:2 FTSA exhibited FXR transactivation and an inhibitory effect through downregulating *HNF4A* gene expression. Interestingly, none of the six PFAS demonstrated significant PPARα transactivation. Furthermore, at the gene expression level, PFOSA, PFPeA, PFPrA, and 6:2 FTSA suppressed *PPARA* gene expression. This study highlights the need for more screening and research on understudied PFAS to understand the toxic underlying mechanism of these PFAS. Based on the results of our study, PFOSA, 6:2 FTSA, and 8:2 FTSA were prioritized for further in-depth studies, as they emerged as the most potent out of the tested PFAS to interfere with lipid metabolism pathways.

## Supplementary Information

Below is the link to the electronic supplementary material.

Supplementary file1 (DOCX 580 KB)

Supplementary file2 (XLSX 42 KB)

Supplementary file3 (XLSX 51 KB)

Supplementary file4 (XLSX 47 KB)

## Data Availability

Raw data are available in the supplements or on request.

## Abbreviations

MTT: 3-(4,5-Dimethyl-thiazol-2-yl)-2,5-diphenyltetrazolium bromide
6: 2 FTOH: 6:2 Fluorotelomer alcohol
6:2 FTSA: 6:2 Fluorotelomer sulfonic acid
8:2 FTSA: 8:2 Fluorotelomer sulfonic acid
AOPs: Adverse outcome pathways
ALT: Alanine aminotransferase
APOA1: Apolipoprotein A1
ABCA1: ATP-binding cassette transporter A1
BA: Bile acid
CSA: Cyclosporine A
CYPRO: Cyproconazole
DMSO: Dimethyl sulfoxide
DMEM: Dulbecco's modified Eagle’s medium
ER: Estrogen receptor
FA: Fatty acid
FBS: Fetal bovine serum
GR: Glucocorticoid receptor
hCAR: Human constitutive androstane receptor
hFXR: Human farnesoid X receptor
hHNF4α: Human hepatocyte nuclear factor-4α
hLXRα: Human liver X receptor alpha
hPXR: Human pregnane X receptor
hRARα: Human retinoic acid receptor-α
HHS: Hydrocortisone hemisuccinate
LDLR: Low-density lipoprotein receptors
NR: Nuclear receptors
ANOVA: One-way analysis of variance
PA + OA: Palmitic acid and oleic acid
P/S: Penicillin and streptomycin
PFAS: Per- and polyfluoroalkyl substances
PFCA: Perfluoroalkyl carboxyl acids
PFSA: Perfluoroalkyl sulfonic acids
PFOSA: Perfluorooctane sulfonamide
PFPeA: Perfluoropentanoic acid
PFPrA: Perfluoropropionic acid
PPREs: Peroxisome proliferator hormone response elements
PPARα: Peroxisome proliferator-activated receptor alpha
PPARẟ: Peroxisome proliferator-activated receptor delta
PPARγ: Peroxisome proliferator-activated receptor gamma
SCARB1: Scavenger receptor class B type 1
SC: Solvent control
SD: Standard deviation
3D: Three-dimensional
TAFLD: Toxicant-associated fatty liver disease
Tyr: Tyrosine
