# Supplementary material for: In vitro screening of understudied PFAS with a focus on lipid metabolism disruption
Source: Arch Toxicol. 2024 Jul 2;98(10):3381–95. doi: 10.1007/s00204-024-03814-2 (PMC11402862; doi:10.1007/s00204-024-03814-2)
Supplement: Supplementary file 1 — Supplementary file1 (DOCX 580 KB) [file 204_2024_3814_MOESM1_ESM.docx]

In vitro Screening of Understudied PFAS with a focus on lipid metabolism disruption

**Lackson Kashobwe^1^, Faezeh Sadrabadi^2^, Albert Braeuning^2^, Pim Leonards^1^, Thorsten Buhrke^2^, and Timo Hamers^1^**

^1^ Vrije Universiteit Amsterdam, Amsterdam Institute for Life and Environment (A-LIFE), De Boelelaan 1085, 1081 HV Amsterdam, the Netherlands. ^2^ German Federal Institute for Risk Assessment (BfR), Department of Food Safety, Max-Dohrn-Str. 8-10, 10589 Berlin, Germany.

Corresponding author: Timo Hamers: timo.hamers@vu.nl

# Supplementary information

**Table S1**. Primers used in this study

| **Gene name** | **Protein name** | **Forward primer (5' – 3')** | **Reverse primer (5’ – 3’)** |
| --- | --- | --- | --- |
| **Marker genes for steatosis** |  |  |  |
| *CCL20* | CCL20 | AGCCCAAGAACAGAAAGAACC | CAAGTCCAGTGAGGCACAAA |
| *CD36* | CD36 | TAGTGACTCTGTGGCTGCTC | CCAGAAGCGACGACATTGAG |
| *FASN* | FASN | ACAGCGGGGAATGGGTACT | GACTGGTACAACGAGCGGAT |
| *INSIG1* | INSIG1 | TGGCAGCTTCCCAAGTATTC | CAAAACTGCGGGTTGGTAAT |
| *SLCO1B1* | SLCO1B1 | ACTGATTCTCGATGGGTTGG | TATTTGGAGTTTGGGGCA |
| *SREBF1* | SREBF1 | CCTGGTCATCTCACAGCAAA | GGCCTTTCACAGAACAGGAA |
| **Bile acid synthesis** |  |  |  |
| *CYP7A1* | CYP7A1 | GACACACCTCGTGGTCCTCT | TTTCATTGCTTCTGGGTTCC |
| *CYP27A1* | CYP27A1 | TTCGAGAAACGCATTGGCTG | GGAGGAAGGTGGCATAGAGTG |
| **Bile acid transport** | | | |
| *ABCB11* | ABCB11 | TAGCCCTGGAGCATTGACAA | CATGGCCACAGTGACGTTAG |
| *ABCC2* | ABCC2 | CAATATCCTGCGCTTTCCCC | CAAGTCATCCCCTCCCAAGT |
| *ABCC3* | ABCC3 | CCGCATCCTGGTTTTAGACG | CAGGTATCAAACTGGGTGCG |
| *NTCP* | NTCP | ATCGTCCTCAAATCCAAACG | TGGCAGAGAGAACTGTGACG |
| *SLCO1B1* | SLCO1B1 | TCCACATCATTTTCAAGGGTCTACT | TGTCTTCATCCATGACACTTCCAT |
| *OSTB* | OSTβ | TGCTGGAAGAGATGCTTTGGT | CTGCTTGCCTGGATGCTTCT |
| **Bile acid detoxification** | | | |
| *BAAT* | BAAT | CCTCATGGCTTGGTACTG | GAGGAGGTGCCACACACTT |
| *UGT1A1* | UGT1A1 | CTGCCTTCACCAAAATCCACTATC | CACAGGACTGTCTGAGGGATTT |
| *UGT2B4* | UGT2B4 | GACAATGGCAAAAGCTGACA | GAGTCCTCCAACGAACTC |
| *SULT2A1* | SULT2A1 | GATCCAATCTGTGCCCATCT | GGGAGGTGGGAGGAGAATAA |
| *CYP3A4* | CYP3A4 | TCACAAACCGGAGGCCTTTT | TGGTGAAGGTTGGAGACAGC |
| **Cholesterol synthesis** | | | |
| *ACAT2* | ACAT2 | ACTTGGCTTACTTGAGAACAGGA | CAGTCAGTGGCATCTCACCTA |
| *SQLE* | SQLE | GCTTCCTTCCTCCTTCATCAGT | AAGCAACAGTCATTCCTCCACC |
| *LCAT* | LCAT | CTCGGCTGTCTACACTTGCT | GCCATCAATAAAGCGGTCCT |
| *SREBF1* | SREBF1 | CGGAACCATCTTGGCAACAGT | CGCTTCTCAATGGCGTTGT |
| **Cholesterol transport** | | | |
| *ABCA1* | ABCA1 | CCTACAGTGATCCCAGCGTG | GGCAGGTACAGCGTGAAGTA |
| *ABCG5* | ABCG5 | TCCTGAGGTTGCCCGATTTG | ATGGACAGCAGAGCCACTAC |
| *ABCG8* | ABCG8 | AAACTTGAGCAGCCTGTGGA | GATGGTGAGGTTCCCGAGAG |
| *SCARB1* | SCARB1 | GGTCCATCTACCCACCCAAC | CAGCGTTGAGGAAGTGAGGAT |
| *LDLR* | LDLR | GCTCCATCGCCTACCTCTTC | TTCTATTGCTGGCCACCTCC |
| **Nuclear receptor** |  |  |  |
| *FXR* | FXR | CAGAGCCAAGGAAGAGATGC | CAATGAGGTGAGGAGGAGGA |
| *PXR* | PXR | GGCATGAAGAAGGAGATGAT | TGGGAGAAGGTAGTGTCAAA |
| *PPARA* | PPARα | GCGAACGATTCGACTCAAGC | CATCCCGACAGAAAGGCACT |
| *PPARD* | PPARδ | GATCCGCATGAAGCTGGAGT | GCTTCCTCTTCTCAGCCTCC |
| *HNF4A* | HNF4α | AGGACTACATCAACGACCGC | ATCTGCTCGATCATCTGCCA |
| Housekeeping genes | | | |
| *GUSB* | GUSB | ACTTCTCTGACAACCGACGC | AGGATCACCTCCCGTTCGTA |
| *GAPDH* | GAPDH | TTAAAAGCAGCCCTGGTGAC | CTCTGCTCCTCCTGTTCGAC |

**Fig S1**. Cytotoxicity profile of the six PFAS based on HEKT293T cell viability. The cells were exposed to different concentrations of 6:2 FTSA, PFPeA, PFPrA, 6:2 FTOH, PFOSA, and 8:2 FTSA for 24 h. The cellular viability was measured using the MTT assay. Viability is shown as a percentage (%) relative to solvent control (SC) set at 100%. Experimental data are the technical replicates' mean, and error bars show the standard deviation (SD).
